# *MADD-FOLH1* Polymorphisms and Their Haplotypes with Serum Lipid Levels and the Risk of Coronary Heart Disease and Ischemic Stroke in a Chinese Han Population

**DOI:** 10.3390/nu8040208

**Published:** 2016-04-08

**Authors:** Dong-Feng Wu, Rui-Xing Yin, Xiao-Li Cao, Feng Huang, Jin-Zhen Wu, Wu-Xian Chen

**Affiliations:** 1 Department of Cardiology, Institute of Cardiovascular Diseases, the First Affiliated Hospital, Guangxi Medical University, 22 Shuangyong Road, Nanning 530021, Guangxi, China; wulove26@tom.com (D.-F.W.); huangfeng3000@126.com (F.H.); wujinzhengx@sohu.com (J.-Z.W.); nncwx@163.com (W.-X.C.); 2 Department of Neurology, the First Affiliated Hospital, Guangxi Medical University, 22 Shuangyong Road, Nanning 530021, Guangxi, China; maten1996@gmail.com

**Keywords:** MAP-kinase activating death domain-folate hydrolase 1 gene, single nucleotide polymorphism, coronary heart disease, ischemic stroke, lipids, interaction

## Abstract

This study aimed to detect the association of the *MADD-FOLH1* single nucleotide polymorphisms (SNPs) and their haplotypes with the risk of coronary heart disease (CHD) and ischemic stroke (IS) in a Chinese Han population. Six SNPs of rs7395662, rs326214, rs326217, rs1051006, rs3736101, and rs7120118 were genotyped in 584 CHD and 555 IS patients, and 596 healthy controls. The genotypic and allelic frequencies of the rs7395662 SNP were different between controls and patients, and the genotypes of the rs7395662 SNP were associated with the risk of CHD and IS in different genetic models. Six main haplotypes among the rs1051006, rs326214, rs326217, rs3736101, and rs7120118 SNPs were detected in our study population, the haplotypes of G-G-T-G-C and G-A-T-G-T were associated with an increased risk of CHD and IS, respectively. The subjects with rs7395662GG genotype in controls had higher triglyceride (TG) and lower high-density lipoprotein cholesterol (HDL-C) levels than the subjects with AA/AG genotypes. Several SNPs interacted with alcohol consumption to influence serum TG (rs326214, rs326217, and rs7120118) and HDL-C (rs7395662) levels. The SNP of rs3736101 interacted with cigarette smoking to modify serum HDL-C levels. The SNP of rs1051006 interacted with body mass index ≥24 kg/m^2^ to modulate serum low-density lipoprotein cholesterol levels. The interactions of several haplotypes and alcohol consumption on the risk of CHD and IS were also observed.

## 1. Introduction

Coronary heart disease (CHD) and ischemic stroke (IS) remain the major causes of morbidity and mortality worldwide [1,2]. The pathological basis of both diseases is atherosclerosis, a progressive inflammatory disorder and, therefore, CHD and IS may share common pathogenesis, as well as many risk factors [2,3]. Previous genome-wide association studies (GWASs) have identified many genes and loci in the predisposition to CHD [4] or IS [5] in different populations. Furthermore, some genetic variants originally shown to influence the risk of CHD were also subsequently found to be associated with IS [6,7].

The MAP-kinase activating death domain (MADD) and folate hydrolase 1 (FOLH1) gene (*MADD-FOLH1*) is located on chromosome 11p. The MADD protein can modulate tumor necrosis factor-alpha and propagate the apoptotic signals [8]. Folate is an essential nutrient that supports nucleotide synthesis and biological methylation reactions. Diminished folate status results in chromosome breakage and is associated with several diseases, including cardiovascular disease [9,10]. Genetic variants involved in folate metabolism may modify the effects of lifestyle (smoking and drinking) on plasma homocysteine concentrations—a risk factor for cardiovascular disease [11]. A novel single nucleotide polymorphism (SNP) of rs7395662 in or near *MADD-FOLH1* has been found association with serum lipid levels [12,13] and the risk of CHD [14] in a previous GWAS and two replication studies. Exactly as patterns of linkage disequilibrium (LD) vary between populations, genetic anomalies of ion channels or specific endothelial regulators may underlie coronary microvascular disease [15,16], the results are complicated and inconsistent across different populations [17,18]. In addition, there are still many variants have not been explored in the *MADD-FOLH1* cluster. Therefore, the purpose of the present study was to detect the association of six SNPs (rs7395662, rs326214, rs326217, rs1051006, rs3736101, and rs7120118) in or near *MADD-FOLH1* and their haplotypes with serum lipid traits and the risk of CHD and IS in a Chinese Han populations.

## 2. Materials and Methods

### 2.1. Study Patients

A total of 1139 unrelated patients with CHD (*n* = 584) and IS (*n* = 555) were recruited from hospitalized patients in the First Affiliated Hospital, Guangxi Medical University. The diagnosis of CHD was based on typical ischemic discomfort plus one or more of electrocardiographic change (ST-segment depression or elevation of ≥0.5 mm, T-wave inversion of ≥3 mm in ≥3 leads, or left bundle branch block), as well as increases in the cardiac markers, including creatinine kinase-MB and troponin T. Coronary angiography was performed in patients with CHD. The coronary angiograms were reviewed by two independent angiographers who were both blinded to the results of the genotypes. For a vessel to be scored, stenosis ≥50% had to be noted in an epicardial coronary vessel of interest or in one of its major branches. In the event of discordance of the number of vessels scored between the two reviewers, angiograms were scored by a third independent reviewer. The selected CHD patients were subject to significant coronary stenosis (≥50%) in at least either one of the three main coronary arteries or their major branches (branch diameter ≥2 mm). Additionally, angiographic severity of disease was classified according to the number of coronary vessels with significant stenosis (luminal narrowing ≥50%) as one-, two-, or three-vessel disease in the three major coronary arteries [19,20]. The diagnosis and classification of IS was ascertained in accordance with the TOAST (Trial of Org 10172 in Acute Stroke Treatment) criteria [21] after strict neurological examination, computed tomography, or magnetic resonance imaging (MRI). The selected IS patients in the study included individuals who were eligible for one of the two subtypes of TOAST criteria: large-artery atherosclerosis and small-vessel occlusion. Individuals with a history of hematologic or brain MRI revealing cerebral hemorrhage, cardioembolic stroke or unspecified stroke, neoplastic or intracranial space-occupying lesion, infection, and other types of intracranial lesions, renal, liver, thyroid, autoimmune diseases, and type 1 diabetes were excluded. The selected IS patients who had a past history of CHD were also excluded, while the selected CHD patients who had a past history of IS were excluded from the study.

### 2.2. Control Subjects

A total of 596 control subjects matched by age, gender, and ethnic group were randomly selected from the healthy adults who underwent periodical medical check-up at the Physical Examination Center of the First Affiliated Hospital, Guangxi Medical University during the same period when CHD and IS patients were recruited. The controls were free of CHD and IS by questionnaires, history-taking, and clinical examination. The examination comprised physical examination, blood sampling, electrocardiography, chest X-ray, and Doppler echocardiography. All enrolled individuals were Han Chinese from Guangxi, the People’s Republic of China. Information on demography, socioeconomic status, medical history, and lifestyle factors was collected by trained research staff with standardized questionnaires for all participants. This study was approved by the Ethics Committee of the First Affiliated Hospital, Guangxi Medical University, and written informed consent was obtained from each participant before data collection. The reported investigations were in accordance with the principles of the Declaration of Helsinki.

### 2.3. Biochemical Measurements

Venous blood samples were obtained from all subjects after at least 12 h of fasting. The levels of serum total cholesterol (TC), triglyceride (TG), high-density lipoprotein cholesterol (HDL-C), and low-density lipoprotein cholesterol (LDL-C) in samples were determined by enzymatic methods with commercially available kits, Tcho-1, TG-LH (RANDOX Laboratories Ltd., Ardmore, Diamond Road, Crumlin Co., Antrim, UK, BT29 4QY), Cholestest N HDL, and Cholestest LDL (Daiichi Pure Chemicals Co., Ltd., Tokyo, Japan), respectively. Serum apolipoprotein (Apo) A1 and ApoB levels were detected by the immunoturbidimetric immunoassay (RANDOX Laboratories Ltd.). All determinations were performed with an autoanalyzer (Type 7170A; Hitachi Ltd., Tokyo, Japan) in the Clinical Science Experiment Center of the First Affiliated Hospital, Guangxi Medical University [22,23,24,25,26,27,28,29,30,31,32,33].

### 2.4. Diagnostic Criteria

The normal values of serum TC, TG, HDL-C, LDL-C, ApoA1, and ApoB levels, and the ratio of ApoA1 to ApoB in our Clinical Science Experiment Center were 3.10–5.17, 0.56–1.70, 0.91–1.81, 2.70–3.20 mmol/L, 1.00–1.78, 0.63–1.14 g/L, and 1.00–2.50, respectively [22,23,24,25,26,27,28,29,30,31,32,33]. Type 2 diabetes was diagnosed according to the WHO diagnostic criteria for diabetes: (1) fasting glucose (FPG) ≥7.0 mmol/L; (2) 2 h postprandial glucose ≥11.1 mmol/L; or (3) self-reported diagnosis of diabetes or use of anti-diabetic medications [34,35]. The individuals with TC >5.17 mmol/L, and/or TG >1.70 mmol/L were defined as hyperlipidemic [36,37,38]. Hypertension was defined according to the criteria outlined by the 1999 World Health Organization—International Society of Hypertension Guidelines for the management of hypertension [39,40,41]. Uncontrolled hypertension was defined as a systolic blood pressure of 140 mmHg or higher and a diastolic blood pressure of 90 mmHg or higher. The subjects with systolic blood pressure of only 140 mmHg or higher but a diastolic blood pressure of <90 mmHg were diagnosed as isolated systolic hypertension. Normal weight, overweight, and obesity were defined as a body mass index (BMI) <24, 24–28, and >28kg/m^2^, respectively [42,43].

### 2.5. SNP Selection

The SNPs were selected on the basis of the following assumptions: (1) Selected SNPs were established by Haploview (Broad Instituteof MIT and Harvard, Cambridge, MA, USA, version 4.2); (2) SNPs information was obtained from NCBI dbSNP Build 132 (http://www.Ncbi.nlm.nih.gov/SNP/); (3) SNPs were restricted to minor allele frequency (MAF) >1%; and (4) SNPs might be associated with the plasma lipid levels or cardiovascular disease in recent studies [12,13,14,15,16,17,18].

### 2.6. Genotyping

Genomic DNA was extracted from leucocytes of venous blood using the phenol-chloroform method, and then sent to the Center for Human Genetics Research, Shanghai Genesky Bio-Tech Co. Ltd. Genotyping of the SNPs were performed by the Snapshot technology platform [22,23,24,25,26,27,28,29,30,31,32,33]. The restriction enzymes for the SNPs were SAP (Promega) and Exonuclease I (Epicentre), respectively. The sense and antisense primers were: rs7120118F: 5′-TGCTCCCCTCTTCCAAACCACT-3′, rs7120118R: 5′-TCCTTCTCCCCAAGACCTCACTC-3′; rs326214F: 5′-TGGCTCATGACAGGTGGTGCTA-3′, rs326214R: 5′-TAGCAGCGGGATGACAGGAAAC-3′; rs326217F: 5′-CCCAGGGACGTTCCTTGTGTAA-3′, rs326217R: 5′-CCTGGTTGCAACATCCACAGAAT-3′; rs7395662F: 5′-CTGTGGCTCCCACATCACTGG-3′, rs7395662R: 5′-AAATGATTTTCCCTGCATGCTAGTT-3′; rs3736101 and rs1051006F: 5′-CGGCCTTTAGGAACCTGCTGAC-3′, rs3736101 and rs1051006R: 5′-TTGGCTGAATCGGGGAGTGTAA-3′.

### 2.7. Statistical Analyses

The statistical analyses were carried out using the statistical software package SPSS 21.0 (SPSS Inc., Chicago, IL, USA). Quantitative variables were expressed as mean ± standard deviation (serum TG levels were presented as medians and interquartile ranges). Qualitative variables were expressed as percentages. Allele frequency was determined via direct counting, and the standard goodness-of-fit test was used to test the Hardy–Weinberg equilibrium. A chi-square analysis was used to evaluate the difference in genotype distribution and sex ratio between the groups. The general characteristics between patient and control groups were tested by the Student’s unpaired *t*-test. The association of genotypes and serum lipid parameters was tested by analysis of covariance (ANCOVA). Any variants associated with the serum lipid parameter at a value of *p* < 0.008 (corresponding to *p* < 0.05 after adjusting for six independent tests by the Bonferroni correction) were considered statistically significant. Unconditional logistic regression was used to assess the correlation between the risk of CHD and IS and genotypes. Age, gender, BMI, smoking, and alcohol consumption were adjusted for the statistical analysis. Odds ratio (OR) and 95% confidence interval (95% CI) were calculated using unconditional logistic regression. The interactions of six SNPs with alcohol consumption, cigarette smoking, BMI ≥ 24 kg/m^2^, age, and sex on serum lipid levels and the risk of CHD and IS were detected by using a factorial regression analysis after controlling for potential confounders, a *P_I_* ≤ 0.0017 was considered statistically significant after Bonferroni correction. The pattern of pair-wise LD between the selected SNPs was measured by *D'* and *r*^2^ using the SHEsis software [44]. Haplotype frequency was determined by means of the algorithms implemented in the PHASE program.

## 3. Results

### 3.1. General Characteristics of the Subjects

The general characteristics of the patients and healthy controls are summarized in Table 1. The values of BMI, pulse pressure, and TG were higher but diastolic blood pressure, TC, HDL-C, ApoA1, the percentages of subjects who consumed alcohol, and the ratio of ApoA1 to ApoB were lower in CHD patients than in controls (*p* < 0.001 for all). The values of BMI, systolic blood pressure, pulse pressure, and TG were higher but TC, HDL-C, ApoA1, the percentages of subjects who consumed alcohol, and the ratio of ApoA1 to ApoB were lower in IS patients than in controls (*p* < 0.001 for all).

### 3.2. Genotypic and Allelic Frequencies in Controls and Patients

The genotypic and allelic frequencies of the 6 *MADD-FOLH1* SNPs are presented in Table 2. The genotype distribution of the 5 SNPs (not including rs1051006 in IS patients, *p* = 0.026) was concordant with the Hardy–Weinberg equilibrium in patients and controls (*p* > 0.05 for all). The genotypic and allelic frequencies of the rs7395662, but not the other five SNPs, were different between controls and patients (CHD and IS, *p* < 0.01 for all), the rs7395662G allele and rs7395662GG genotype frequencies were lower in CHD (G, 36.6%; GG, 12.7%) or IS (G, 36.3%; GG, 14.4%) patients than in control subjects (G, 42.6%; GG, 18.8%; *p* < 0.01 for all).

### 3.3. Genotypes of the Six MADD-FOLH1 SNPs and the Risk of CHD and IS

As shown in Table 3, the genotypes of the rs7395662, but not the other five SNPs, were associated with the risk of CHD after the Bonferroni correction (a value of *p* < 0.008 was considered statistically significant) in different genetic models: co-dominant model: GG *vs.* AA (OR = 0.78, 95% CI = 0.66–0.92, *p* = 0.0068); recessive model: GG *vs.* AA/GA (OR = 0.63, 95% CI = 0.46–0.86, *p* = 0.0038); and log-additive model: G *vs.* A (OR = 0.78, 95% CI = 0.66–0.92, *p* = 0.003).

The genotypes of the rs7395662 SNP were also associated with the risk of IS in different genetic models: dominant model: GA/GG *vs.* AA (OR = 0.70, 95% CI = 0.55–0.89, *p* = 0.0039); and log-additive model: G *vs.* A (OR = 0.78, 95% CI = 0.66–0.91, *p* = 0.0024).

### 3.4. Haplotypes and the Risk of CHD and IS

There was strong LD among the rs1051006, rs326214, rs326217, rs3736101, and rs7120118 SNPs in controls and patients (*D'* = 0.8946–0.9983) but weak LD between the rs7395662 and the other five SNPs (*D'* = 0.1127–0.3275). Thus, haplotype analyses among the five SNPs and the associations of their haplotypes and the risk of CHD and IS were performed. Six main haplotypes are shown in Table 4. The haplotype of G-G-T-G-C (in the order of rs1051006, rs326214, rs326217, rs3736101 and rs7120118 SNPs) was associated with an increased risk for CHD (adjusted OR = 1.59, 95% CI = 1.06–2.38, *p* = 0.026). The haplotype of G-A-T-G-T was associated with an increased risk for IS (adjusted OR = 1.95, 95% CI = 1.04–3.68, *p* = 0.039).

### 3.5. Genotypes and Serum Lipid Levels

The association of the *MADD-FOLH1* SNPs and serum lipid levels in controls is presented in Table 5. Serum TG and HDL-C levels were different among the three genotypes of the rs7395662 but not the other five SNPs (*p =* 0.005 and 0.001; respectively), the subjects with rs7395662GG genotype had higher TG and lower HDL-C levels than the subjects with rs7395662AA and rs7395662AG genotypes. There was no difference in serum TC, LDL-C, ApoA1, ApoB levels, and the ApoA1/ApoB ratio among the three genotypes of the SNP.

### 3.6. Interactions of the MADD-FOLH1 SNPs and Drinking, Smoking, BMI, Age, and Sex on Serum Lipid Levels and the Risk of CHD and IS

**Figure 1:**
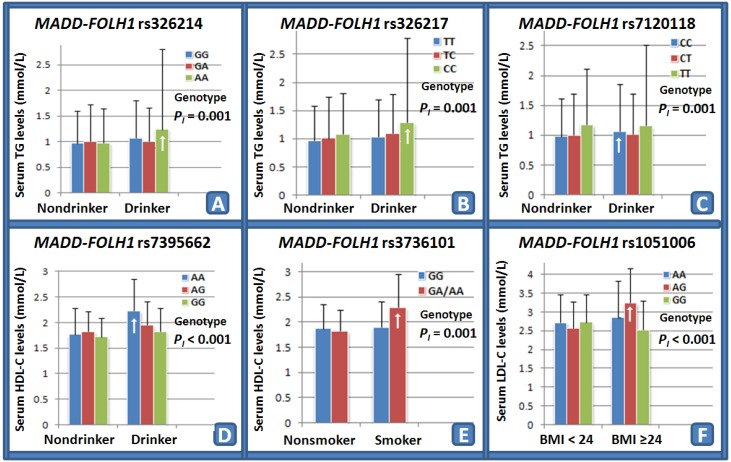
Interactions of the *MADD-FOLH1* SNPs and drinking, smoking, BMI, age, and sex on serum lipid levels. MADD-FOLH1, MAP-kinase activating death domain-folate hydrolase 1; TG, triglyceride; HDL-C, high-density lipoprotein cholesterol; LDL-C, low-density lipoprotein cholesterol; BMI, body mass index. The differences in serum TG levels among the genotypes were determined by the Kruskal–Wallis test. The differences in serum HDL-C and LDL-C levels among the genotypes were assessed using analysis of covariance. The interactions of the genotypes and alcohol consumption, cigarette smoking or BMI ≥ 24 kg/m^2^ on serum lipid levels were detected by using a factorial regression analysis after controlling for potential confounders (*P_I_*). Genotype and alcohol consumption, cigarette smoking or BMI ≥ 24 kg/m^2^ interaction increases serum lipid levels (↑). A *P_I_* ≤ 0.0017 was considered statistically significant after Bonferroni correction.

The interactions of the *MADD-FOLH1* SNPs and drinking, smoking, BMI, age, and sex on serum lipid levels and the risk of CHD and IS are shown in Table 6. Several SNPs interacted with alcohol consumption to influence serum TG (rs326214, Figure 1A; rs326217, Figure 1B; and rs7120118, Figure 1C) and HDL-C (rs7395662, Figure 1D) levels. The SNP of rs3736101 interacted with cigarette smoking to modify serum HDL-C (Figure 1E) levels. The SNP of rs1051006 interacted with BMI ≥ 24 kg/m^2^ to modulate serum LDL-C (Figure 1F) levels.

### 3.7. Interactions of the Genotypes and Drinking, Smoking, and BMI on the Risk of CHD and IS

The rs3736101GA/AA genotypes interacted with alcohol consumption to decrease the risk of CHD (OR = 0.33, 95% CI = 0.12–0.89, *p* = 0.0021). No interactions of the genotypes and smoking and BMI on the risk of CHD and IS was detected in our study population.

### 3.8. Interactions of the Haplotypes and Drinking on the Risk of CHD

The interactions of several haplotypes and alcohol consumption on the risk of CHD were noted in this study. As compared with the A-G-T-G-C haplotype in non-drinkers, the haplotypes of G-G-T-A-C (OR = 1.69, 95% CI = 1.02–2.81) and G-A-T-G-T (OR = 3.61, 95% CI = 1.29–10.15) in non-drinkers were associated with an increased risk for CHD, whereas the haplotypes of A-G-T-G-C (OR = 0.34, 95% CI = 0.22–0.51), G-A-C-G-T (OR = 0.39, 95% CI = 0.25–0.59), G-G-T-A-C (OR = 0.11, 95% CI = 0.04–0.30) and G-A-C-G-C (OR = 0.17, 95% CI = 0.07–0.40) in drinkers were associated with a decreased risk for CHD.

For the drinkers, as compared with the A-G-T-G-C haplotype, the haplotype of G-G-T-G-C (OR = 2.38, 95% CI = 1.25–4.56) was associated with an increased risk for CHD, whereas the haplotype of G-G-T-A-C (OR = 0.32, 95% CI = 0.12–0.87) was associated with a decreased risk for CHD.

As compared with the same haplotype in non-drinkers, the haplotypes of A-G-T-G-C (OR = 0.34, 95% CI = 0.22-0.51), G-A-C-G-T (OR = 0.36, 95% CI = 0.24–0.54), G-G-T-A-C (OR = 0.06, 95% CI = 0.02–0.19), G-A-C-G-C (OR = 0.18, 95% CI = 0.06–0.53), and G-A-T-G-T (OR = 0.07, 95% CI = 0.01–0.34) in drinkers were associated with a decreased risk for CHD.

### 3.9. Interactions of the Haplotypes and Drinking on the Risk of IS

The interactions of several haplotypes and alcohol consumption on the risk of IS were also noted in this study. As compared with the A-G-T-G-C haplotype in non-drinkers, the haplotype of G-A-T-G-T (OR = 4.05, 95% CI = 1.46–11.27) in non-drinkers was associated with an increased risk for IS, whereas the haplotypes of A-G-T-G-C (OR = 0.33, 95% CI = 0.22–0.50), G-A-C-G-T (OR = 0.39, 95% CI = 0.26–0.58), G-G-T-A-C (OR = 0.41, 95% CI = 0.22–0.79), G-A-C-G-C (OR = 0.25, 95% CI = 0.12–0.52), and G-A-T-G-T (OR = 0.28, 95% CI = 0.10–0.79) in drinkers were associated with a decreased risk for IS.

For the drinkers, as compared with the A-G-T-G-C haplotype, no significant association was detected between the haplotypes and IS.

As compared with the same haplotype in non-drinkers, the haplotypes of A-G-T-G-C (OR = 0.33, 95% CI = 0.22–0.50), G-A-C-G-T (OR = 0.48, 95% CI = 0.32–0.72), G-G-T-A-C (OR = 0.30, 95% CI = 0.13–0.67), G-A-C-G-C (OR = 0.37, 95% CI = 0.14–0.98), and G-A-T-G-T (OR = 0.07, 95% CI = 0.02–0.29) in drinkers were associated with a decreased risk for IS.

## 4. Discussion

In the present study, we showed that the genotypic and allelic frequencies of the rs7395662 SNP were different between controls and CHD or IS patients, and that the rs7395662 genotypes or alleles were associated with the risk of CHD and IS in different genetic models. The SNPs of rs1051006, rs326214, rs326217, rs3736101, and rs7120118 were strong LD in controls and patients. Six main haplotypes of the five SNPs were detected. The haplotype of G-G-T-G-C was associated with an increased risk for CHD, whereas the haplotype of G-A-T-G-T was associated with an increased risk for IS. The subjects with rs7395662GG genotype in controls had higher TG and lower HDL-C levels than the subjects with rs7395662AA and rs7395662AG genotypes. Several SNPs interacted with alcohol consumption to influence serum TG (rs326214, rs326217, and rs7120118) and HDL-C (rs7395662) levels. Two SNPs interacted with cigarette smoking to modify serum TG (rs7395662) and HDL-C (rs3736101) levels. The SNP of rs1051006 interacted with BMI ≥ 24 kg/m^2^ to modulate serum LDL-C levels. The rs3736101GA/AA genotypes interacted with alcohol consumption to decrease the risk of CHD. The interactions of several haplotypes and alcohol consumption on the risk of CHD and IS were also observed. To the best of our knowledge, this is the first report to evaluate the interaction between the six *MADD-FOLH1* SNPs and their haplotypes and several environmental factors on serum lipid levels and the risk of CHD and IS.

We showed that the genotypic and allelic frequencies of the rs7395662, but not the other five SNPs were different between controls and CHD or IS patients, the patients with CHD (36.6%, *p* = 0.003) or IS (36.3%, *p* = 0.002) had lower frequencies of rs7395662G allele than the controls (42.6%). The genotypes of the rs7395662 SNP were also associated with the risk of CHD and IS after the Bonferroni correction in different genetic models. In a previous GWAS, the rs7395662G allele frequency was 61% in a total of 17,797–22,562 persons, aged 18-104 years and from geographic regions spanning from the Nordic countries to Southern Europe [12]. In two recent studies, we and other researchers showed that the allelic frequency of rs7395662G was 44.9% in the healthy Mulao population [13], 43.7% in Han Chinese [13], 47.3% in CHD cases [14], and 51.9% in non-CHD controls [14]. The data in the International HapMap Project’s database have suggested that the frequency of rs7395662G allele was 62.7% in European, 58.9% in Han Chinese in Beijing, 48.9% in Japanese, and 44.2% in Sub-Saharan African [13]. As compared with the other populations, we found that the frequency of rs7395662G allele in our study populations was lower than that in Han Chinese from Beijing, which may be caused by different sample sizes and Han Chinese from Beijing and Guangxi are different parts of Han. These results suggest that the prevalence of the rs7395662G allele variation may have racial/ethnic- and sex-specificity. The prevalence of the rs7395662G allele is higher in European than in Chinese. These findings may also partly explain why the prevalence of cardiovascular disease is different between European and Chinese.

The association of the rs1051006, rs326214, rs326217, rs3736101, and rs7120118 SNPs and the risk of CHD and IS has not been reported previously. In the present study, we showed that there were no associations of the five SNPs and the risk of CHD and IS. However, there was high LD among the rs1051006, rs326214, rs326217, rs3736101, and rs7120118 SNPs in controls and patients. Haplotype analyses of the five SNPs showed that the haplotype of G-G-T-G-C was associated with an increased risk for CHD, whereas the haplotype of G-A-T-G-T was associated with an increased risk for IS. However, these findings still need to be confirmed in the other populations with larger sample sizes.

The results of the present study showed that serum TG and HDL-C levels were different among the three genotypes of the rs7395662, but not the other five SNPs, the subjects with rs7395662GG genotype had higher TG and lower HDL-C levels than the subjects with rs7395662AA and rs7395662AG genotypes. There was no difference in serum TC, LDL-C, ApoA1, ApoB levels, and the ApoA1/ApoB ratio among the three genotypes of the SNP. The association between the rs7395662 SNP and serum or plasma lipid levels has been investigated in several previous studies [12,13,14]. In a previous GWAS, Aulchenko *et al.* [12] showed significant association between the rs7395662 SNP and HDL-C levels in 16 population-based cohorts (*p* = 6 × 10^−11^). The coded allele (the allele for which effect was estimated) was the rs7395662G allele. In a previous comparative study of two ethnic groups, we found that there may be a sex-specific association of the rs7395662 SNP and serum lipid concentrations in the Mulao and Han populations [13]. A recent study also showed that the rs7395662A allele was significantly associated with decreased HDL-C levels (*β* = −0.024, *p* = 0.007) in 1069 healthy control subjects [14]. However, the association of the rs1051006, rs326214, rs326217, rs3736101, and rs7120118 SNPs and serum or plasma lipid phenotypes is not well-known. In a previous study, Akiyama *et al.* [8] investigated functional genes at homologous loci identified using human lipid GWASs that responded to a high-fat, high-cholesterol diet intervention in an animal model. They showed that the gene of *MADD* rs7120118 SNP was potential target of lipid association (HDL-C). *MADD* encodes the MADD protein, which interacts with tumor necrosis factor-alpha receptor 1 to activate mitogen-activated protein kinase and propagate apoptotic signals [8]. In addition, G protein-coupled receptor kinase 5, which is also reported to interact with MADD [45], has been demonstrated to exhibit a significant increase in its gene expression induced by high-fat, high-cholesterol diet intervention, supporting the potential involvement of both G protein-coupled receptor kinase 5 and MADD in lipid metabolism [8].

The interactions of the *MADD-FOLH1* SNPs and their haplotypes and some environmental factors on serum lipid levels and the risk of CHD and IS are not known. In the present study, we firstly showed that several *MADD-FOLH1* SNPs interacted with alcohol consumption to influence serum TG (rs326214, rs326217, and rs7120118) and HDL-C (rs7395662) levels. The SNP of rs3736101 interacted with cigarette smoking to modify serum HDL-C levels. The SNP of rs1051006 interacted with BMI ≥ 24 kg/m^2^ to modulate serum LDL-C levels. The haplotypes of G-G-T-A-C and G-A-T-G-T in non-drinkers were associated with an increased risk for CHD, whereas the haplotypes of A-G-T-G-C, G-A-C-G-T, G-G-T-A-C, and G-A-C-G-C in drinkers were associated with a decreased risk for CHD. The haplotype of G-A-T-G-T in non-drinkers was associated with an increased risk for IS, whereas the haplotypes of A-G-T-G-C, G-A-C-G-T, G-G-T-A-C, G-A-C-G-C, and G-A-T-G-T in drinkers were associated with a decreased risk for IS. It is well known that heavy alcohol intake and cigarette smoking have a disadvantageous effect on lipid profiles. In the current study, however, we showed that the rs3736101 AG/GG genotypes interacted with cigarette smoking to increase serum HDL-C levels. The reason for this contradictory finding is not clear. In our study populations, most smokers also have drinking habits. Thus, these interactions still need to be determined.

There were several potential limitations in this study. Firstly, the sample size was relatively small compared to many GWASs and replication studies. Therefore, further studies with larger sample sizes are needed to confirm our results. Secondly, there were significant differences in the general characteristics between the control and patient groups. Although age, gender, BMI, cigarette smoking, and alcohol consumption have been adjusted for the statistical analysis, we could not completely eliminate the potential effects of these factors on serum lipid levels and the risk of CHD and IS. Thirdly, the association of the six SNPs and serum lipid levels in the CHD and IS groups was not analyzed because of the interference of lipid-lowering drugs. Finally, it is well known that both CHD and IS are the complex multifactorial disorders that are believed to result from an interaction between the genetic background of an individual and various environmental factors. Although we have detected the association between six *MADD-FOLH1* SNPs and their haplotypes and the risk of CHD and IS, there are still many unmeasured environmental and genetic factors and their interactions.

## 5. Conclusions

The results of the present study showed that the genotypic and allelic frequencies of the rs7395662 SNP were different between controls and patients; the rs7395662 genotypes were associated with the risk of CHD and IS in different genetic models. Six main haplotypes among the rs1051006, rs326214, rs326217, rs3736101, and rs7120118 SNPs were detected. The haplotype of G-G-T-G-C was associated with an increased risk for CHD, whereas the haplotype of G-A-T-G-T was associated with an increased risk for IS. The subjects with rs7395662GG genotype in controls had higher TG and lower HDL-C levels than the subjects with rs7395662AA and rs7395662AG genotypes. Several SNPs and their haplotypes interacted with alcohol consumption, cigarette smoking, and BMI ≥ 24 kg/m^2^ to modify serum TG, HDL-C, and LDL-C levels, and the risk of CHD and IS.

## Figures and Tables

**Table 1:** Comparison of general characteristics and serum lipid levels between controls and patients.

Parameter	Control	CHD	IS	*P_1_*	*P_2_*
Number	596	584	555	–	–
Male/Female	435/161	432/152	401/154	0.701	0.780
Age, years	61.53 ± 10.88	62.21 ± 10.50	62.82 ± 12.35	0.275	0.061
Body mass index, kg/m^2^ (kg/m^2^)	22.42 ± 2.86	23.84 ± 3.82	23.40 ± 3.51	0.000	0.000
Systolic blood pressure, mmHg	130.63 ± 20.02	132.98 ± 23.31	147.72 ± 22.15	0.066	0.000
Diastolic blood pressure, mmHg	83.00 ± 13.42	79.18 ± 14.22	83.78 ± 12.97	0.000	0.332
Pulse pressure, mmHg	49.78 ± 14.70	53.42 ± 18.03	63.78 ± 18.30	0.000	0.000
Cigarette smoking, *n* (%)	267 (44.8)	284 (48.6)	248 (44.68)	0.187	0.969
Alcohol consumption, *n* (%)	284 (47.7)	169 (28.9)	172 (31.0)	0.000	0.000
Total cholesterol, mmol/L	4.94 ± 1.11	4.53 ± 1.19	4.52 ± 1.15	0.000	0.000
Triglyceride, mmol/L	1.01 (0.71)	1.36(0.96)	1.36(0.93)	0.000	0.000
HDL-C, mmol/L	1.90 ± 0.50	1.14 ± 0.34	1.23 ± 0.40	0.000	0.000
LDL-C, mmol/L	2.74 ± 0.80	2.71 ± 1.00	2.68 ± 0.90	0.532	0.263
Apolipoprotein (Apo) A1, g/L	1.41 ± 0.28	1.04 ± 0.52	1.02 ± 0.22	0.000	0.000
ApoB, g/L	0.91 ± 0.22	0.91 ± 0.27	0.89 ± 0.25	0.987	0.349
ApoA1/ApoB	1.63 ± 0.50	1.23 ± 0.79	1.26 ± 0.60	0.000	0.000

CHD, coronary heart disease; IS, ischemic stroke; HDL-C, high-density lipoprotein cholesterol; LDL-C, low-density lipoprotein cholesterol. The value of triglyceride was presented as median (interquartile range), the difference between CHD/IS patients and controls was determined by the Wilcoxon–Mann–Whitney test. *P_1_*, CHD *vs.* controls; *P_2_*, IS *vs.* controls.

**Table 2:** Genotypic and allelic frequencies of six SNPs in controls and patients (*n* (%)).

SNP/Genotype	Control *n* = 596	CHD *n* = 584	IS *n* = 555	Allele	Control	CHD	IS
**rs7395662**							
AA	200 (33.6)	231 (39.6)	232 (41.8)				
AG	284 (47.7)	279 (47.8)	243 (43.8)	A	684 (57.4)	741 (63.4)	707 (63.7)
GG	112 (18.8)	74 (12.7)	80 (14.4)	G	508 (42.6)	427 (36.6)	403 (36.3)
χ^2^		9.917	9.445	χ^2^		9.054	9.574
*P*		0.007	0.009	*P*		0.003	0.002
*P_HWE_*	0.530	0.470	0.210				
**rs326214**							
GG	311 (52.1)	295 (50.4)	291 (52.4)				
GA	243 (40.7)	248 (42.4)	233 (42.0)	G	865 (72.4)	838 (71.6)	815 (73.4)
AA	43 (7.2)	42 (7.2)	31 (5.6)	A	329 (27.6)	332 (28.4)	295 (26.6)
χ^2^		0.363	1.291	χ^2^		0.198	0.279
*P*		0.834	0.524	*P*		0.656	0.598
*P_HWE_*	0.633	0.299	0.074				
**rs326217**							
TT	326 (54.6)	313 (53.5)	316 (56.9)				
TC	232 (38.9)	234 (40.0)	213 (38.4)	T	884 (74.0)	860 (73.5)	845 (76.2)
CC	39 (6.5)	38 (6.5)	26 (4.7)	C	310 (26.0)	310 (26.5)	264 (23.8)
χ^2^		0.164	2.038	χ^2^		0.087	1.431
*P*		0.921	0.361	*P*		0.769	0.232
*P_HWE_*	0.791	0.515	0.188				
**rs1051006**							
AA	242 (40.5)	207 (35.5)	213 (38.4)				
AG	275 (46.1)	293 (50.3)	281 (50.6)	A	759 (63.6)	707 (60.6)	707 (63.7)
GG	80 (13.4)	83 (14.2)	61 (11.0)	G	435 (36.4)	459 (39.4)	403 (36.4)
χ^2^		3.188	2.946	χ^2^		2.157	0.004
*P*		0.203	0.229	*P*		0.142	0.950
*P_HWE_*	0.893	0.203	0.026				
**rs3736101**							
GG	543 (91.0)	526 (90.2)	495 (89.2)				
GA	52 (8.7)	55 (9.4)	60 (10.8)	G	1138 (95.3)	1107 (94.9)	1050 (94.6)
AA	2 (0.3)	2 (0.3)	0	A	56 (4.7)	59 (5.1)	60 (5.4)
χ^2^		0.188	3.264	χ^2^		0.174	0.616
*P*		0.910	0.196	*P*		0.676	0.433
*P_HWE_*	0.529	0.662	0.178				
**rs7120118**							
CC	342 (57.5)	317 (54.3)	315 (56.8)				
CT	224 (37.6)	232 (39.7)	216 (38.9)	C	908 (76.3)	866 (74.1)	846 (76.2)
TT	29 (4.9)	35 (6.0)	24 (4.3)	T	282 (23.7)	302 (25.9)	264 (23.8)
χ^2^		1.549	0.336	χ^2^		1.474	0.002
*P*		0.461	0.845	*P*		0.225	0.961
*P_HWE_*	0.317	0.383	0.083				

SNP, single nucleotide polymorphism; CHD, coronary heart disease; IS, ischemic stroke; HWE, Hardy-Weinberg equilibrium.

**Table 3:** Genotypes of the six *MADD-FOLH1* SNPs and the risk of CHD and IS.

SNP/Model	Ref. Genotype	Effect Genotype	CHD (OR 95% CI)	*p*	IS (OR 95% CI)	*p*
**rs7395662**						
Co-dominant	AA	GA	0.85 (0.66–1.09)	0.0068	0.74 (0.57–0.95)	0.0088
		GG	0.78 (0.66–0.92)		0.62 (0.44–0.87)	
Dominant	AA	GA/GG	0.77 (0.61–0.98)	0.032	0.70 (0.55–0.89)	0.0039
Recessive	AA/GA	GG	0.63 (0.46–0.86)	0.0038	0.73 (0.53–1.00)	0.046
Overdominant	AA/GG	GA	1.00 (0.80–1.26)	0.97	0.86 (0.68–1.08)	0.19
Log-additive			0.78 (0.66–0.92)	0.003	0.78 (0.66–0.91)	0.0024
**rs326214**						
Co-dominant	GG	GA	1.08 (0.85–1.37)	0.83	1.02 (0.81–1.30)	0.52
		AA	1.03 (0.65–1.62)		0.77 (0.47–1.26)	
Dominant	GG	GA/AA	1.07 (0.85–1.34)	0.57	0.99 (0.78–1.24)	0.91
Recessive	GG/GA	AA	1.00 (0.64–1.55)	0.99	0.76 (0.47–1.23)	0.26
Overdominant	GG/AA	GA	1.07 (0.85–1.35)	0.56	1.05 (0.83–1.33)	0.66
Log-additive			1.04 (0.87–1.25)	0.65	0.95 (0.79–1.15)	0.59
**rs326217**						
Co-dominant	TT	TC	1.05 (0.83–1.33)	0.92	0.95 (0.74–1.21)	0.36
		CC	1.01 (0.63–1.63)		0.69 (0.41–1.16)	
Dominant	TT	CT/CC	1.05 (0.83–1.31)	0.7	0.91 (0.72–1.15)	0.43
Recessive	TT/CT	CC	0.99 (0.63–1.58)	0.98	0.70 (0.42–1.17)	0.17
Overdominant	TT/CC	CT	1.05 (0.83–1.32)	0.69	0.98 (0.77–1.24)	0.87
Log-additive			1.03 (0.85–1.24)	0.77	0.89 (0.74–1.08)	0.24
**rs1051006**						
Co-dominant	AA	GA	1.25 (0.97–1.60)	0.20	1.16 (0.91–1.49)	0.23
		GG	1.21 (0.85–1.74)		0.87 (0.59–1.27)	
Dominant	AA	GA/GG	1.24 (0.98–1.57)	0.075	1.09 (0.86–1.39)	0.45
Recessive	AA/GA	GG	1.07 (0.77–1.49)	0.68	0.80 (0.56–1.14)	0.21
Overdominant	AA/GG	GA	1.18 (0.94–1.49)	0.15	1.20 (0.95–1.51)	0.12
Log-additive			1.14 (0.96–1.34)	0.14	0.99 (0.84–1.18)	0.95
**rs3736101**						
Co-dominant	GG	GA	1.09 (0.73–1.63)	0.91	1.27 (0.86–1.87)	0.13
		AA	1.03 (0.14–7.36)		0.00 (0.00–NA)	
Dominant	GG	GA/AA	1.09 (0.74–1.61)	0.67	1.22 (0.83–1.80)	0.32
Recessive	GG/GA	AA	1.02 (1.14–7.29)	0.98	0.00 (0.00–NA)	0.1
Overdominant	GG/AA	GA	1.09 (0.73–1.62)	0.67	1.27 (0.86–1.88)	0.23
Log-additive			1.08 (0.75–1.57)	0.68	1.16 (0.80–1.70)	0.43
**rs7120118**						
Co-dominant	CC	CT	1.12 (0.88–1.42)	0.46	1.05 (0.82–1.33)	0.85
		TT	1.30 (0.78–2.18)		0.90 (0.51–1.58)	
Dominant	CC	CT/TT	1.14 (0.90–1.43)	0.27	1.03 (0.82–1.30)	0.8
Recessive	CC/CT	TT	1.24 (0.75–2.06)	0.40	0.88 (0.51–1.53)	0.66
Overdominant	CC/TT	CT	1.09 (0.86–1.38)	0.46	1.06 (0.83–1.34)	0.66
Log-additive			1.13 (0.93–1.37)	0.22	1.01 (0.82–1.23)	0.96

SNP, single nucleotide polymorphism; CHD, coronary heart disease; IS, ischemic stroke.

**Table 4:** Haplotype frequencies of the five *MADD-FOLH1* SNPs and the risk of CHD and IS.

Haplotype	Control Frequency	CHD			IS		
		Frequency	OR (95% CI)	*p*	Frequency	OR (95% CI)	*p*
A-G-T-G-C	0.6339	0.6178	1.00		0.6332	1.00	
G-A-C-G-T	0.2199	0.2281	1.07 (0.86–1.33)	0.54	0.2161	0.91 (0.73–1.13)	0.38
G-G-T-G-C	0.0419	0.0511	1.59 (1.06–2.38)	0.026	0.0439	1.06 (0.69–1.64)	0.78
G-G-T-A-C	0.0469	0.0492	1.12 (0.74–1.68)	0.59	0.0505	1.30 (0.87–1.94)	0.20
G-A-C-G-C	0.0397	0.0329	0.75 (0.45–1.27)	0.28	0.0337	0.71 (0.43–1.17)	0.18
G-A-T-G-T	0.0159	0.0177	1.74 (0.88–3.46)	0.11	0.0202	1.95 (1.04–3.68)	0.039

CHD, coronary heart disease; IS, ischemic stroke. The haplotypes consist of five alleles in the order of rs1051006, rs326214, rs326217, rs3736101, and rs7120118 SNPs.

**Table 5:** Genotypes of the six *MADD-FOLH1* SNPs and serum lipid levels in controls.

SNP/Genotype	*n*	TC (mmol/L)	TG (mmol/L)	HDL-C (mmol/L)	LDL-C (mmol/L)	ApoA1 (g/L)	ApoB (g/L)	ApoA1/ApoB
**rs7395662**								
AA	200	5.00 ± 1.06	1.03 (0.58)	1.99 ± 0.61	2.79 ± 0.83	1.44 ± 0.29	0.92 ± 0.23	1.65 ± 0.57
AG	284	4.88 ± 0.94	0.98 (0.64)	1.88 ± 0.44	2.75 ± 0.76	1.39 ± 0.24	0.90 ± 0.19	1.61 ± 0.41
GG	112	5.00 ± 1.46	1.23 (0.79)	1.77 ± 0.42	2.64 ± 0.81	1.36 ± 0.23	0.90 ± 0.23	1.62 ± 0.49
*F*	–	0.846	10.554	7.482	1.313	3.929	1.143	0.525
*P*	–	0.430	0.005	0.001	0.270	0.020	0.320	0.592
**rs326214**								
GG	311	4.98 ± 1.15	1.01 (0.71)	1.90 ± 0.54	2.75 ± 0.83	1.40 ± 0.30	0.90 ± 0.22	1.64 ± 0.54
GA	243	4.90 ± 0.91	1.01 (0.67)	1.93 ± 0.48	2.76 ± 0.76	1.40 ± 0.20	0.81 ± 0.21	1.61 ± 0.42
AA	43	4.97 ± 1.56	1.18 (0.75)	1.71 ± 0.26	2.63 ± 0.79	1.35 ± 0.20	0.87 ± 0.24	1.63 ± 0.45
*F*	–	0.360	3.771	3.654	0.486	0.959	0.730	0.225
*P*	–	0.698	0.115	0.026	0.616	0.384	0.482	0.799
**rs326217**								
TT	326	4.97 ± 1.14	1.00 (0.65)	1.91 ± 0.55	2.74 ± 0.83	1.41 ± 0.29	0.90 ± 0.22	1.65 ± 0.53
TC	232	4.90 ± 0.91	1.01 (0.71)	1.90 ± 0.47	2.80 ± 0.75	1.39 ± 0.20	0.92 ± 0.20	1.60 ± 0.41
CC	39	5.02 ± 1.61	1.23 (0.75)	1.74 ± 0.26	2.60 ± 0.78	1.36 ± 0.21	0.87 ± 0.25	1.65 ± 0.46
*F*	–	0.323	5.090	2.273	0.913	0.701	0.736	0.691
*P*	–	0.724	0.078	0.104	0.402	0.497	0.480	0.501
**rs1051006**								
AA	242	5.02 ± 1.12	1.03 (0.67)	1.92 ± 0.55	2.76 ± 0.80	1.41 ± 0.28	0.91 ± 0.21	1.62 ± 0.52
AG	275	4.86 ± 1.01	1.01 (0.71)	1.88 ± 0.46	2.75 ± 0.81	1.40 ± 0.24	0.91 ± 0.22	1.62 ± 0.46
GG	80	5.00 ± 1.28	1.02 (0.74)	1.87 ± 0.50	2.71 ± 0.74	1.38 ± 0.22	0.88 ± 0.21	1.65 ± 0.47
*F*		1.512	0.244	0.600	0.110	0.453	0.889	0.112
*P*		0.221	0.885	0.549	0.896	0.636	0.411	0.894
**rs3736101**								
GG	543	4.94 ± 1.11	1.02 (0.66)	1.88 ± 0.49	2.74 ± 0.80	1.39 ± 0.25	0.91 ± 0.22	1.61 ± 0.47
GA/AA	54	5.02 ± 0.96	1.00 (0.79)	2.02 ± 0.60	2.82 ± 0.79	1.45 ± 0.29	0.88 ± 0.20	1.76 ± 0.64
*F*		0.307	0.214	3.487	0.518	2.333	1.156	4.530
*P*		0.580	0.831	0.062	0.472	0.127	0.283	0.034
**rs7120118**								
CC	342	4.97 ± 1.14	1.02 (0.73)	1.90 ± 0.54	2.75 ± 0.82	1.41 ± 0.30	0.90 ± 0.22	1.64 ± 0.53
CT	224	4.88 ± 0.88	1.01 (0.69)	1.90 ± 0.47	2.75 ± 0.75	1.39 ± 0.18	0.91 ± 0.20	1.61 ± 0.41
TT	29	5.15 ± 1.86	1.18 (0.88)	1.76 ± 0.28	2.66 ± 0.87	1.33 ± 1.33	0.89 ± 0.30	1.61 ± 0.49
*F*		0.927	1.035	1.188	0.202	1.576	0.072	0.383
*P*		0.396	0.596	0.306	0.817	0.208	0.931	0.682

SNP, single nucleotide polymorphism; TC, total cholesterol; TG, triglyceride; HDL-C, high-density lipoprotein cholesterol; LDL-C, low-density lipoprotein cholesterol; ApoA1, apolipoprotein A1; ApoB, apolipoprotein B. The value of triglyceride was presented as median (interquartile range), and the difference among the genotypes was determined by the Kruskal-Wallis test. A *p <* 0.008 was considered statistically significant after Bonferroni correction.

**Table 6:** The *P_I_* values for interactions of genotypes and drinking, smoking, and BMI on serum lipid levels and the risk of CHD and IS.

SNP/Factor	Lipid							CHD	IS
	TC	TG	HDL-C	LDL-C	ApoA1	ApoB	ApoA1/B		
rs7395662									
Drinking	0.175	0.002	0.000	0.454	0.064	0.314	0.073	0.620	0.650
Smoking	0.031	0.002	0.128	0.037	0.789	0.046	0.046	0.630	0.590
BMI	0.314	0.991	0.367	0.222	0.530	0.687	0.711	0.602	0.453
Age	0.004	0.003	0.635	0.425	0.390	0.277	0.709	0.141	0.019
Sex	0.377	0.106	0.118	0.852	0.345	0.272	0.077	0.344	0.178
rs326214									
Drinking	0.611	0.001	0.212	0.282	0.393	0.309	0.196	0.380	0.420
Smoking	0.686	0.016	0.121	0.118	0.366	0.626	0.755	0.419	0.475
BMI	0.735	0.131	0.792	0.133	0.130	0.100	0.792	0.432	0.341
Age	0.145	0.004	0.762	0.948	0.703	0.334	0.583	0.225	0.865
Sex	0.506	0.020	0.582	0.335	0.951	0.718	0.506	0.596	0.842
rs326217									
Drinking	0.529	0.001	0.192	0.307	0.295	0.392	0.089	0.440	0.072
Smoking	0.854	0.015	0.438	0.259	0.521	0.808	0.967	0.909	0.900
BMI	0.917	0.084	0.670	0.056	0.094	0.042	0.670	0.503	0.334
Age	0.321	0.004	0.844	0.799	0.716	0.141	0.439	0.398	0.821
Sex	0.510	0.207	0.726	0.577	0.890	0.921	0.718	0.680	0.678
rs1051006									
Drinking	0.780	0.019	0.914	0.449	0.817	0.886	0.798	0.539	0.181
Smoking	0.456	0.065	0.180	0.040	0.468	0.715	0.338	0.348	0.614
BMI	0.028	0.432	0.321	0.000	0.116	0.011	0.941	0.120	0.430
Age	0.019	0.031	0.082	0.697	0.366	0.107	0.573	0.153	0.679
Sex	0.718	0.097	0.975	0.096	0.645	0.360	0.605	0.995	0.608
rs3736101									
Drinking	0.881	0.297	0.002	0.649	0.028	0.179	0.152	0.0021	0.650
Smoking	0.911	0.450	0.001	0.375	0.075	0.853	0.376	0.630	0.550
BMI	0.064	0.537	0.099	0.198	0.758	0.779	0.427	0.649	0.881
Age	0.986	0.374	0.942	0.632	0.859	0.152	0.244	0.218	0.383
Sex	0.073	0.233	0.396	0.343	0.720	0.099	0.169	0.467	0.758
rs7120118									
Drinking	0.287	0.001	0.359	0.887	0.198	0.430	0.063	0.590	0.640
Smoking	0.881	0.013	0.423	0.103	0.348	0.159	0.543	0.489	0.391
BMI	0.323	0.020	0.786	0.216	0.421	0.032	0.865	0.604	0.567
Age	0.102	0.002	0.817	0.771	0.288	0.781	0.958	0.258	0.780
Sex	0.234	0.055	0.963	0.785	0.746	0.493	0.749	0.505	0.491

SNP, single nucleotide polymorphism; TC, total cholesterol; TG, triglyceride; HDL-C, high-density lipoprotein cholesterol; LDL-C, low-density lipoprotein cholesterol; ApoA1, apolipoprotein A1; ApoB, apolipoprotein B; CHD, coronary heart disease; IS, ischemic stroke; BMI, body mass index. A *P_I_* ≤ 0.0017 was considered statistically significant after Bonferroni correction.

## Abbreviations

The following abbreviations are used in this manuscript:

ANCOVA	Analysis of covariance
Apo	Apolipoprotein
CHD	Coronary heart disease
CI	Confidence interval
FOLH1	Folate hydrolase 1
FPG	Fasting glucose
GWASs	Genome-wide association studies
HDL-C	High-density lipoprotein cholesterol
IS	Ischemic stroke
LD	Linkage disequilibrium
LDL-C	Low-density lipoprotein cholesterol
MADD	MAP-kinase activating death domain
MAF	Minor allele frequency
MRI	Magnetic resonance imaging
OR	Odds ratio
SNP	Single nucleotide polymorphism
TC	Total cholesterol
TG	Triglyceride
